# Down-Regulation of FcεRI-Mediated CD63 Basophil Response during Short-Term VIT Determined Venom-Nonspecific Desensitization

**DOI:** 10.1371/journal.pone.0094762

**Published:** 2014-04-14

**Authors:** Nina Čelesnik Smodiš, Mira Šilar, Renato Eržen, Matija Rijavec, Mitja Košnik, Peter Korošec

**Affiliations:** University Clinic of Respiratory and Allergic Diseases Golnik, Golnik, Slovenia; Ludwig-Maximilians-University Munich, Germany

## Abstract

**Background:**

We recently showed a desensitization of FcεRI-mediated basophil response after short-term VIT. Our aim was to evaluate the allergen specificity of this desensitization.

**Methods:**

In 11 *Hymenoptera*-venom double positive subjects, basophil threshold sensitivity (CD-sens) to anti-FcεRI, honeybee, and *Vespula* venom was assessed at the beginning and just before the first maintenance dose (MD) of single ultra-rush VIT. In some patients we also monitored CD-sens to rApi m 1 and/or rVes v 5 or other co-sensitizations (i.e., grass pollen). In additional 7 patients, basophils were stripped and sensitized with house dust mite (HDM) IgEs at the same time points.

**Results:**

We demonstrated a marked reduction of CD-sens to anti-FcεRI and VIT-specific venom before the first MD in all 18 subjects included. Furthermore, in 10 out of 11 double positive subjects, a significant and comparable decrease before the first MD was also evident for non-VIT venom; this nonspecific decrease was further supported by the opposite recombinant species-specific major allergen. In one subject with additional grass pollen allergy, a decrease of CD-sens to grass allergen was also demonstrated. Similarly, in 7 cases of patients with passively HDM-sensitized basophils, a significant reduction of CD-sens was also evident to *de novo* sensitized HDM allergen.

**Conclusions:**

Short-term VIT induced basophil desensitization to VIT-specific as well as to VIT-nonspecific venom. As opposed to long-term VIT, which induces venom-specific changes, the effect of short-term VIT seems to be venom-nonspecific.

## Introduction

Venom immunotherapy (VIT) is unambiguously the treatment of choice for prevention of severe systemic allergic reactions induced by *Hymenoptera* stings [1]–[3] and the early protective mechanisms that lead to unresponsiveness to the sensitizing allergen seem to develop during the course of short-term VIT, as soon as the maintenance dose (MD) is achieved [4]–[6]. Despite its effectiveness, the precise immunological mechanisms for the immediate protection of VIT have not yet been explained. Recently we showed that short-term VIT induced a marked desensitization of FcεRI-mediated basophil activation and that this desensitization was evident in both adults and children before the first MD, within 5 days of ultra-rush or a few weeks of semi-rush VIT, but not during the buildup phase [7]. These changes were comparable with other reports that demonstrated decreased IgE receptor-induced histamine, sulfidoleukotriene, and cytokine release during short-term VIT [8], [10], [11]. Unfortunately, these studies did not clarify the allergen specificity of this desensitization (i.e., if these changes are also relevant for possible non-VIT co-sensitizing allergens beyond VIT allergen), as shown previously by means of decreased peripheral leukocyte sensitivity to mediator release during short-term immunotherapy to the allergen injected and other unrelated sensitizing allergen not included in the therapy [12]–[15]. Similar nonspecific effect was found during short-term *in vitro* subthreshold basophil desensitization [16], [17].

For this reason, we carried out a complex follow-up study to evaluate basophil threshold sensitivity to anti-FcεRI, VIT, and non-VIT venom in double positive adult subjects at the beginning and just before the first MD of single ultra-rush VIT. In some patients we also monitored basophil sensitivity to opposite, non-VIT venom major allergens such as rVes v 5 or rApi m 1 or other co-sensitizing aeroallergens. To further assess whether these changes were cellular-based, we set up a controlled experimental design of a passive IgE sensitization of stripped honeybee (HBV) or *Vespula* venom (VV) basophils. Thus, at the beginning and just before the first MD, the patients' basophils were isolated and sensitized with house dust mite (HDM) serum IgE antibodies and followed up for basophil threshold sensitivity to HDM allergen. Finally, all patients were monitored for whole blood FcεRI gene and basophil cell-surface expression.

## Materials and Methods

### Study population

Eleven subjects (mean age 41 years; range 23–55; 10 men) with double positive sIgE and basophil activation test (BAT) to HBV and VV were included in this prospective study (Table 1). Double positivity to HBV and VV was confirmed in nine (1–3, 6–11) with clinical history and recombinant Api m 1 and Ves v 5 or v 1 by sIgE and/or BAT. Subjects nos. 4 and 5 were positive only to rVes v 5, but also had a clear history of anaphylaxis after honeybee sting (Mueller grade IV and I, respectively) and double positive sIgE, skin, and/or BAT to both venoms [18]. The clinical relevance of an additional grass pollen allergy in subject no. 11 was confirmed by sIgE, skin test, BAT, and recombinant major allergens Phl p 1, 5b. For the passive IgE sensitization experiment, seven subjects (39 years; 23–56; 4 male) with single positive clinical history, venom-specific IgE and/or BAT and species-specific recombinants to HBV or VV were included in the study (Table 2). They all had negative sIgE, skin test, and BAT to HDM allergen.

**Table 1:** Clinical data, sIgE and BAT in double positive subjects.

Patient no.	Age (years)	Sex	Mueller grade		Initial VIT	sIgE (kUA/L)		Diagnostic BAT (%)		sIgE (kUA/L)		
			HB	V		HBV	VV	HBV	VV	rApi m1	rVes v5/v1	OSR
						(i1)	(i3)	1/0.1 µg/mL	1/0.1 µg/mL	(i208)	(i209/i211)	(f316)
1	49	M	IV	LLR	HBV	13.0	1.23	97/41	56/3	1.95	1.46	1.63
2	26	M	III	II	HBV	5.24	2.23	94/73	67/3	0.94	0.66	<0.35
3	54	M	III	LLR	HBV	14.0	2.41	70/46	65/5	2.65	0.84	0.67
4	47	M	IV	I	HBV	1.71	1.12	95/77	72/7	<0.35	0.37	<0.35
5	36	M	I	IV	VV	2.26	8.14	56/5	85/56	<0.35	2.27	2.35
6	31	M	III	II	HBV	4.41	0.81	96/84	94/61	0.86	1.07	<0.35
7	55	M	nk	IV	VV	4.59	0.70	73/42	73/19	1.04	<0.35	<0.35
8	31	M	I	IV	VV	9.94	25.0	72/4	71/6	0.35	4.45	11.2
9	55	F	II	nk	HBV	3.50	0.36	94/43	76/2	1.21	0.38	<0.35
10	23	M	III	LLR	HBV	9.74	5.00	82/74	66/18	3.25	1.97	3.70
11*	40	M	III	LLR	HBV	10.7	3.06	94/93	96/28	0.59	0.49	3.86

M: male, F: female, HB(V): Honeybee (venom), V(V): *Vespula* (venom).

LLR: large local reaction, nk: the degree of reaction after the sting is not known.

Diagnostic BAT: the threshold value for diagnostically positive results was defined as 15% of CD63-positive basophils [20]–[22].

*Patient with additional grass pollen sensitization: skin prick test (mixed grasses; HAL Allergy) pos; sIgE Timothy (g6): 31.1 kUA/L; rPhl p 1,5b (g213): 9.15 kUA/L.

All sIgE were measured with ImmunoCAP-FEIA (Phadia, Thermo Fisher Scientific, Uppsala, Sweden).

**Table 2:** Clinical data, sIgE and BAT in subjects for passive IgE sensitization.

Patient no.	Age (years)	Sex	Mueller grade	VIT	sIgE (kUA/L)		Diagnostic BAT (%)		sIgE (kUA/L)		
					HBV	VV	HBV	VV	rApi m1	rVes v5/v1	OSR
					(i1)	(i3)	1/0.1 µg/mL	1/0.1 µg/mL	(i208)	(i209/i211)	(f316)
12	40	F	II (HB)	HBV	0.36	<0.35	73/78	10/6	<0.35	<0.35	<0.35
13	55	F	III (V)	VV	<0.35	12.3	6/3	72/64	<0.35	69.20	<0.35
14	35	M	IV (HB)	HBV	11.6	1.47	96/86	5/2	0.49	<0.35	3.14
15	56	M	IV (V)	VV	<0.35	1.17	3/1	76/4	<0.35	0.36	<0.35
16	23	F	III (V)	VV	<0.35	21.9	14/3	51/4	<0.35	1.81	<0.35
17	32	M	III (HB)	HBV	1.33	<0.35	89/29	36/2	4.01	<0.35	0.49
18	33	M	III (V)	VV	<0.35	4.21	14/10	77/8	<0.35	1.14	<0.35

M: male, F: female, HB(V): Honeybee (venom), V(V): *Vespula* (venom).

Diagnostic BAT: the threshold value for diagnostically positive results was defined as 15% of CD63-positive basophils [20]–[22].

All sIgE were measured with ImmunoCAP-FEIA (Phadia, Thermo Fisher Scientific, Uppsala, Sweden).

In the double positive group, the venom for initial ultra-rush VIT was selected according to greater severity of induced anaphylaxis (5 Mueller grade IV, 5 III, and 1 II), and therefore eight subjects were enrolled in initial ultra-rush VIT with HBV and three with VV (Table 1). From the single positive group, three subjects with Mueller grades IV, III and II were enrolled in HBV and one with grade IV and three with grade III in VV ultra-rush VIT (Table 2). The ultra-rush *Hymenoptera* VIT protocol was performed as previously described in detail [7]. Blood samples were taken before the beginning of treatment and at the end of the buildup phase, before the first MD administration at day 5. All subjects gave written informed consent and the study was approved by the Slovenian National Medical Ethics Committee.

### Basophil threshold sensitivity and CD-sens calculation

A basophil activation assay was performed on the heparinized whole blood incubated with basophil stimulation buffer with IL-3 (Bühlmann, Switzerland) containing serial dilutions of anti-FcεRI mAbs (550–0.55 ng/ml; Bühlmann), HBV (1–0.001 µg/ml) and VV (1–0.001 µg/ml; Hal Allergie, Netherlands) or rApi m 1 (8–0.08 µg/ml) and/or rVes v 5 (1–0.001 µg/ml) expressed in *E. coli* [19] or grass pollen (10–0.1 µg/mL; AQUAGEN SQ, grass pollen mix L299, ALK Abello, Spain), at 37 °C for 15 minutes. Degranulation was stopped by chilling on ice, after which anti-CD63, anti-CD123, and anti-HLA-DR mAb (BD Biosciences, USA) were added and incubated for 20 minutes. Finally, whole blood probes were lysed, washed, fixed, and analyzed within 2 hours on a FACSCalibur flow cytometer (BD Biosciences) [7], [20]–[22].

Basophil sensitivity was determined as the allergen concentration giving a 50% of maximum CD63% up-regulation. CD sensitivity (CD-sens) was calculated as the inverse value of this threshold allergen concentration multiplied by 100, as previously demonstrated [7], [23]–[25]. The higher value for CD-sens represents higher basophil sensitivity.

### Passive IgE sensitization

Peripheral blood mononuclear cells (PBMCs) from heparinized blood of HBV or VV single positive donors were first separated by Ficoll-Paque (GE Healthcare Bio-Sciences AB, Sweden) density gradient and washed twice with RPMI 1640 (GIBCO, USA) and again with RPMI 1640 with 0.5% BSA (Sigma, USA; RPMI-BSA). PBMCs were then treated with lactic acid buffer (pH 3.9) containing 13.4 mM lactic acid, 140 mM NaCl, and 5 mM KCl (Sigma) for 3.5 minutes, and with 15 µl PBS-TrisBase (ImmunoConcepts, USA) and washed again with RPMI-BSA. The cells were re-suspended in a solution containing sera from subjects allergic to HDM allergen, 32 µl 0.1 M EDTA (Sigma), and 8 µl heparin 250 IU/ml (Krka, Slovenia), and placed in a CO_2_ incubator for 90 min at 37 °C. Afterwards the cells were washed, re-suspended in RPMI-BSA, and immediately used in BAT, where they were stimulated with serial dilutions of *D. pteronyssinus* (333.3–1.665 ng/ml; Bühlmann). The BAT protocol was completely the same as for heparinized whole blood samples [7], [20]–[22].

### FcεRI gene expression profiles

Whole blood FceRI gene expression levels were determined as previously described [7]. Briefly, total RNA was isolated from whole blood using the PAXgene Blood miRNA Kit (PreAnalytiX GmbH, Switzerland) and quantified by Qubit fluorometer (Invitrogen Corporation, USA). Following reverse transcription, cDNA was quantified by real-time PCR (ABI PRISM 7500 Real-Time PCR System) at standard conditions using TaqMan Universal PCR Master Mix (Applied Biosystems, USA). Expression levels of the α-subunit of high-affinity IgE receptor (*FCER1A*) (Hs00175232_m1) were normalized against ribosomal 18s RNA Endogenous Control (Applied Biosystems). All measurements were performed in triplicate for each sample and time point and relative expression were analyzed using the ΔΔCt method.

### FceRI cell-surface expression

The number of FceRI receptors per basophil (CD123+ HLA-DR– cells) was analyzed using a FITC-conjugated antibody to FceRI (eBioscience, USA) and standard curve of Calibration Beads (Dako Cytomation, Denmark) as previously described [7], [24].

### Absolute basophil cell count

For the absolute basophil count (CD123+ HLA-DR– cells) we added AccuCount Fluorescent microbeads (Spherotec Inc., USA) to fixed samples before analysis. The absolute number of basophils per µl of whole blood was calculated using the following equation: (number of events for basophil region/number of events for microbeads region) × (number of microbeds used in test/volume of the whole blood sample initially used) as previously described [7].

### Statistical analyses

Depending on the distribution of the data, we used either a Wilcoxon matched pairs test or a paired *t*-test. Data were expressed as median (IQR) or mean (95% CI). Probability values (*P*) of less than 0.05 were accepted as significant. Analyses were performed using GraphPad Prism 5.

## Results

### Basophil threshold sensitivity (CD-sens) to VIT and non-VIT venom

**Figure 1:**
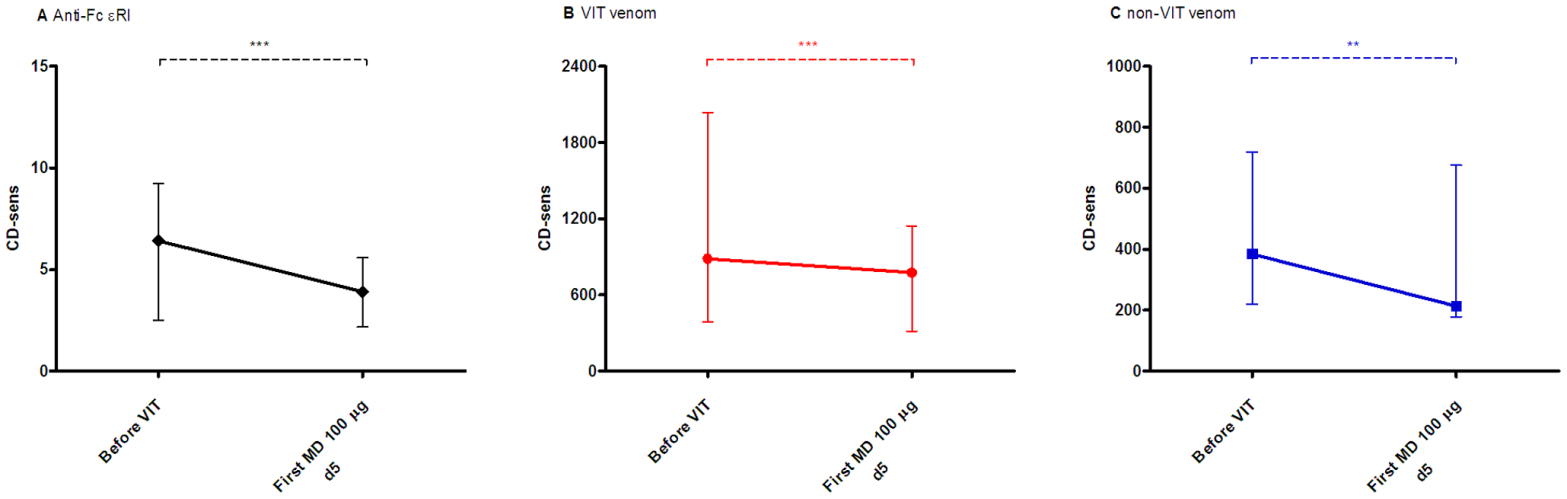
A–C. Basophil threshold sensitivity (CD-sens). Basophil threshold sensitivity (CD-sens) in 11 double and 7 single positive subjects in **A** to anti-FcεRI and in **B** to VIT venom and in 11 double positive subjects in **C** to non-VIT venom stimulation before treatment and the first maintenance dose of single ultra-rush VIT. Data are presented as median values with interquartile range. **P<.01 ***P<.001.

A marked reduction of CD-sens to anti-FcεRI and VIT-specific venom was demonstrated before the first MD in comparison to before VIT in all subjects included (before VIT: median 6.4, IQR 2.5–9.3 and 884, 389–2034 vs. before MD: 3.9, 2.2–5.6 and 775, 312–1143, respectively; *P*<.001; Figure 1A, B). Furthermore, a significant and comparable decrease was also evident in non-VIT venom in 10 out of 11 double positive subjects (385, 219–719 vs. 213, 178–676, *P*<.01; Figure 1C).

### Basophil response to recombinant species-specific major allergens

**Figure 2:**
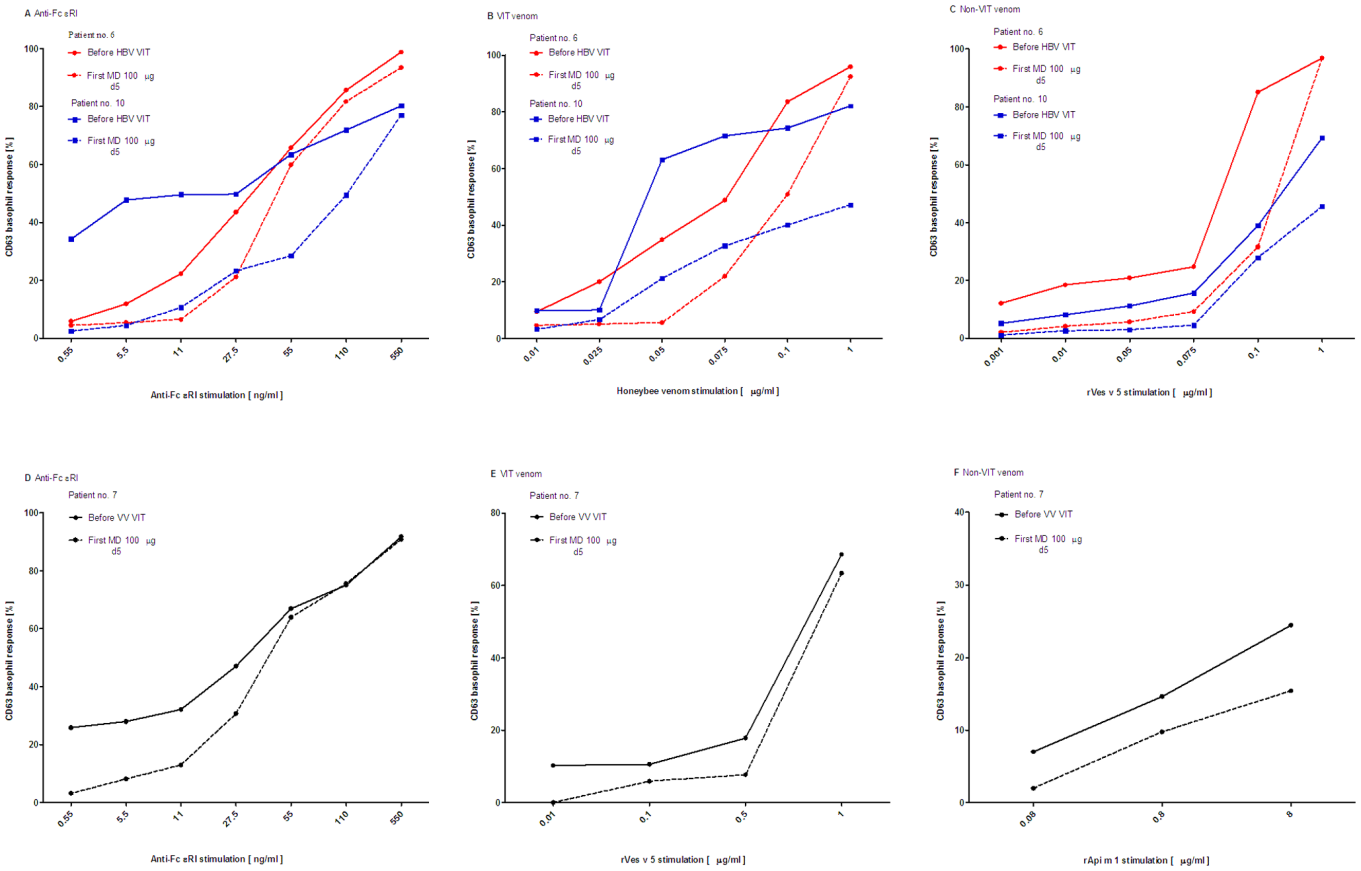
A–F. Basophil response to recombinant species-specific major allergens. CD63 basophil dose-response curves in double positive patients nos. 6 and 10 in **A** to anti-FcεRI in **B** to honeybee and in **C** to rVes v 5 stimulation before treatment and the first maintenance dose of honeybee ultra-rush VIT and in double positive patient no. 7 in **D** to anti-FcεRI in **E** to rVes v 5 and in **F** to rApi m 1 stimulation before treatment and the first maintenance dose of *Vespula* ultra-rush VIT.

To determine that the observed basophil desensitization to non-VIT venom is not related to cross-reactive epitopes, we included the opposite recombinant species-specific major allergens. Patients nos. 6 and 10 were followed at the beginning and before the first MD of HBV VIT. CD-sens to anti-FcεRI and HBV markedly decreased before the first MD when compared to before HBV VIT (before VIT: 2.3; 1538 and 41.1; 524 vs. before MD: 2.1; 1042 and 1.4; 322, separately; Figure 2A, B). Moreover, a comparable decrease was evident in response to non-VIT rVes v 5 allergen (1136 and 621 vs. 877 and 535, separately; Figure 2C). Patient no. 7 was monitored during VV VIT. Similarly, the decrease in CD-sens before the first MD versus the state before VV VIT was evident to anti-FcεRI (before VIT: 4.0 vs. before MD: 2.6), rVes v 5 (144 vs. 122) and non-VIT rApi m 1 (152 vs. 132), shown in Figure 2D–F.

### Basophil response in patient co-sensitized to grass pollen allergen

**Figure 3:**
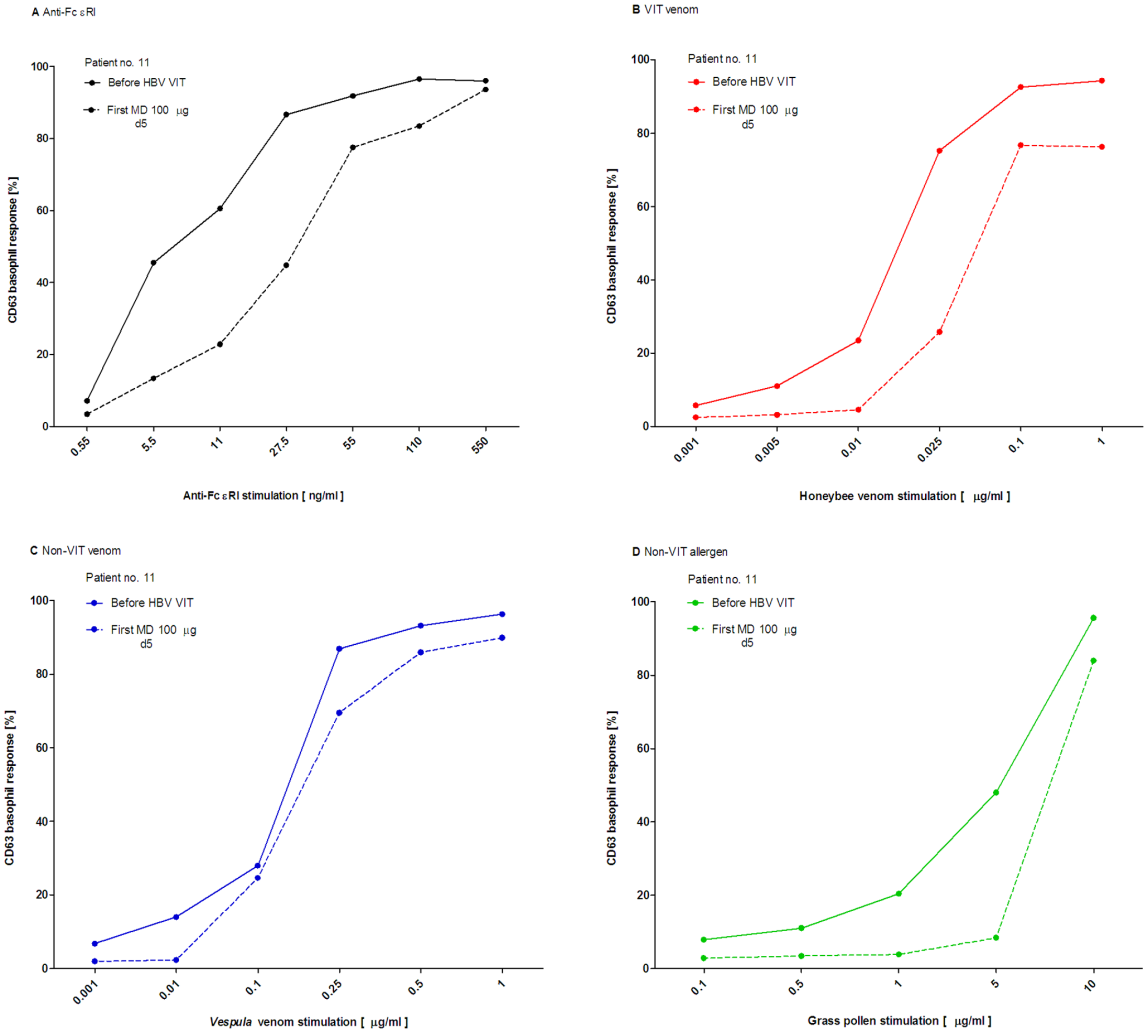
A–D. Basophil response in patient co-sensitized to grass pollen allergen. CD63 basophil dose-response curve in poly-sensitized patient no. 11 in **A** to anti-FcεRI in **B** to honeybee in **C** to *Vespula* venom and in **D** to grass pollen stimulation before treatment and the first maintenance dose of honeybee ultra-rush VIT.

To further clarify the allergen specificity of basophil desensitization, patient no. 11 suffering from allergic rhinitis was followed before treatment and the first MD of HBV VIT. An evident reduction in CD-sens was demonstrated before the first MD in comparison to before HBV VIT to anti-FcεRI (before VIT: 15.6 vs. before MD: 3.9) and all co-sensitizing allergens i.e., HBV (6667 vs. 3448), VV (719 vs. 676) and also to grass pollen (20.5 vs. 12.1), demonstrated in Figure 3A–D.

### Passive IgE sensitization

**Figure 4:**
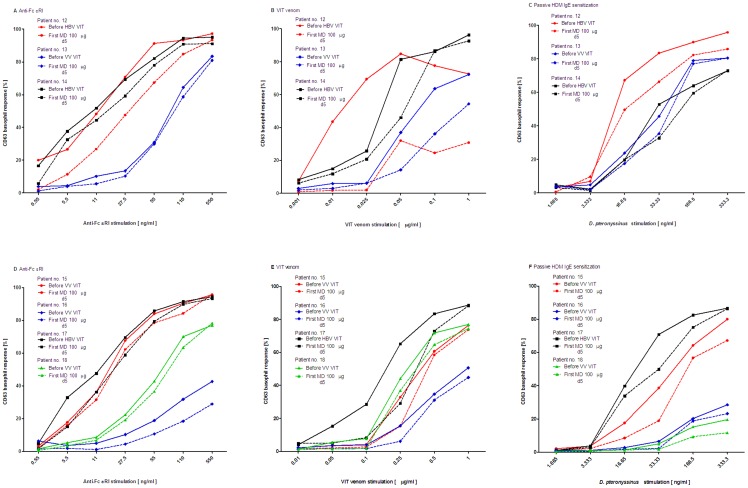
A–F. Passive IgE sensitization (dose-response curves). CD63 basophil dose-response curves in patients nos. 12–18 in **A** and **D** to anti-FcεRI in **B** and **E** to VIT venom and in **C** and **F** after passive IgE sensitization of stripped basophils also to house dust mite stimulation, all before treatment and the first maintenance dose of ultra-rush VIT.

**Figure 5:**
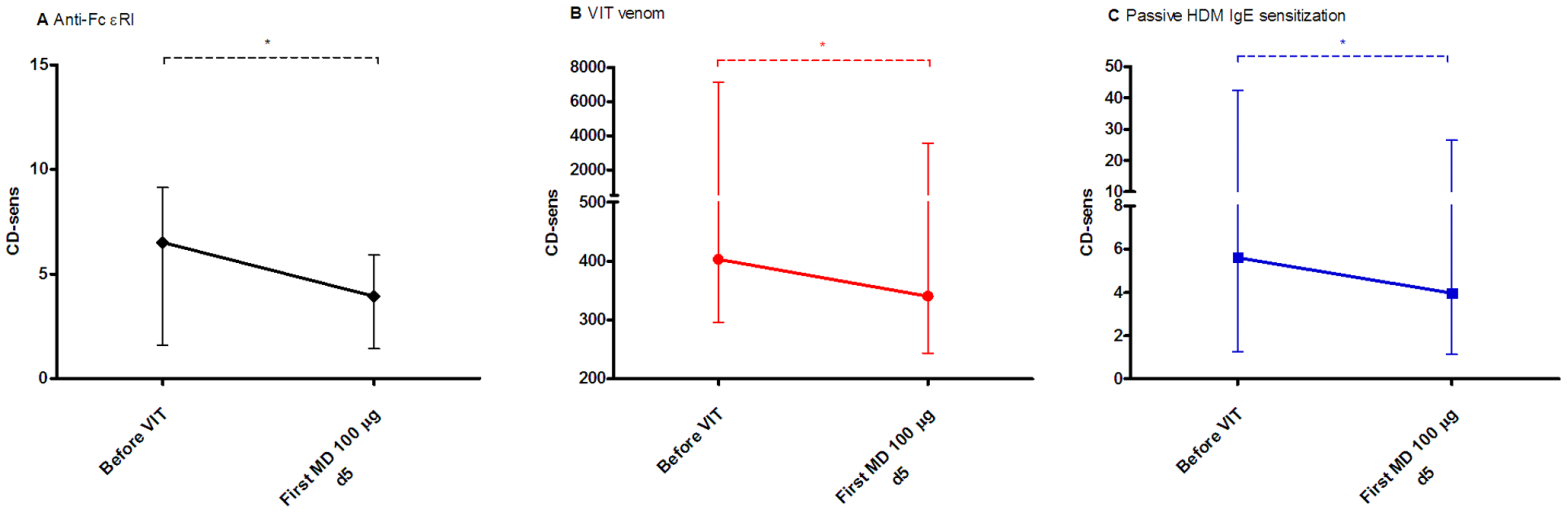
A–C. Passive IgE sensitization (CD-sens). CD-sens in in patients nos. 12–18 in **A** to anti-FcεRI in **B** to VIT venom and in **C** after passive IgE sensitization of stripped basophils also to house dust mite stimulation before treatment and the first maintenance dose of ultra-rush VIT. Data are presented as median values with interquartile range. *P<.05.

To further assess whether these basophil changes were cellular-based, we conducted a controlled experiment of passive IgE sensitization of 7 HBV or VV stripped basophils with sera of HDM allergic subjects before treatment and the first MD of VIT. All individual CD63 basophil dose-response curves to anti-FcεRI, VIT-specific venom and to *de novo* sensitized HDM allergen were decreased at the level of the first MD when compared to before VIT (patients nos. 12–18; Figure 4A–F). CD-sens of HBV (patients nos. 12, 14 and 17) and VV allergic subjects (patient nos. 13, 15, 16 and 18) before the first MD was significantly decreased when compared to before VIT to anti-FcεRI (before VIT: median 6.5, IQR 1.6–9.2 vs. before MD: 3.9, 1.4–5.9; *P*<.05; Figure 5A) and to VIT-specific venom (403, 296–7143 vs. 340, 244–3571; *P*<.05; Figure 5B). Moreover, after passive IgE sensitization of those stripped basophils with HDM-specific IgEs, a similar reduction was found to *de novo* sensitized HDM allergen. CD-sens to HDM stimulation at the level of the first MD versus before VIT was significantly decreased (before VIT nos. 12–18: 5.6, 1.3–42 vs. 3.9, 1.1–26; *P*<.05; Figure 5C).

### FcεRI gene and cell-surface expression

We found a reduced expression of FcεRI gene before the first MD of VIT versus before treatment in 11 of 18 subjects included (61%) (before VIT mean −0.040, 95% CI −0.654 to 0.574 vs. before MD: −0.174, −0.868 to 0.520); however, the differences were not significant. A similar reduction was also evident in basophil FcεRI cell-surface expression (84×10^3^ mol per cell, 64−104×10^3^ vs. 80×10^3^, 61−100×10^3^ in 8 of 13 subjects included (62%); *P*>.05).

### Absolute basophil cell count

The blood basophil numbers before the first MD were highly comparable to prior to treatment (before VIT mean 23 basophils per µl; 95% CI 18–28 vs. before MD: 24; 17–31).

## Discussion

We recently showed that short-term VIT induced a marked desensitization of FcεRI-mediated basophil response [7] and hereinafter we seek to determine the allergen specificity of this desensitization. In the current study, before the first MD within 5 days of ultra-rush VIT, we demonstrated the basophil desensitization to anti-FcεRI and to VIT-specific as well as to VIT-nonspecific venom. In limited number of patients this nonspecific basophil desensitization was further supported by opposite venom recombinant species-specific major allergen and also in case of other co-sensitizations (i.e., grass pollen). Furthermore, the venom-nonspecific base of these changes was demonstrated in the controlled experimental design of stripped patients' basophils passively sensitized with HDM IgEs and stimulated with HDM.

It has been shown that VIT is effective as soon as the MD is achieved [4], [5], and in the case of an ultra-rush protocol this is in a timeframe of a few days; however, for induction of long-lasting protection (i.e., tolerance), the treatment should last at least 3 to 5 years [1]–[3], [26]. The precise mechanisms of action for both the quickly established protection and the induction of long-term tolerance have not yet been explained, despite different approaches that followed the action from blocking IgGs up to Treg cells [6], [8], [11], [26]–[28]. Recent reports have focused on the basophils. Induction of tolerance in adults was demonstrated to be significantly associated with an approximately fourfold decrease in basophil response to VIT-venom submaximal stimulation [21]. Similar results were shown in children after 6 months and 2 to 4 years of VIT [20]. The mechanisms responsible for basophil suppression after long-term or VIT withdrawal is unknown: first, it seems that these changes are not related to the blocking role of sIgGs [21], [29], as shown in the pollen immunotherapy model [30], [31] and, second, those studies did not show any alternations in basophil response to non-VIT co-sensitizing allergens [20], [21].

Unlike the long-term action, knowledge about the early protective changes after short-term VIT became more evident with two recent reports and also current data, clearly showing a prompt desensitization of FcεRI-mediated basophil activation either by up-regulation of histamine receptor 2 or by down-regulation of FcεRI expression [7], [9]. These FcεRI-pathway-mediated alternations obviously suggest that early events might be cellular-based and thus not only relevant for VIT-venom response, as demonstrated previously [8], [11], [32]. For this reason, we followed double positive subjects during initial single short-term VIT and found basophil desensitization to both VIT and non-VIT venom. This nonspecific desensitization was also confirmed by non-VIT venom major recombinant allergens (rVes v 5 and rApi m 1) or co-sensitizing aeroallergens and thus is not very likely to be related to cross-reactive epitopes. Furthermore, to better show the cellular-based desensitization, patients' basophils were stripped for IgEs, sensitized with HDM-specific IgEs, and stimulated with HDM allergen. Similarly, we found a significant reduction of CD-sens to *de novo* sensitized HDM allergen. Furthermore this experiment on washed basophils indicates that different humoral factors from sera or plasma-like different antibodies or cytokines-may not be critical for early basophil desensitization [6], [8], [27], [28], [30], [31]. Beside decreased mediator release, a depletion of circulating effectors cells has been proposed as a potential early protective mechanism [8], [11]. Pierkes at al. observed reduced basophil numbers at the maintenance level, 1 week after the beginning of treatment [8], while most studies depicted reductions only during buildup [7], [9], [10], [33] with return to pretreatment baseline values at the maintenance level, within 1–2 weeks or 6 months after the beginning of treatment [7], [10], [33], which is in concordance with our findings. The discrepancies observed could be an issue of different samplings and timeframes between immunotherapy protocols applied (rush or semi-rush vs. ultra-rush), thus also affecting the dynamic cellular turnover of basophils [34].

What was important in the analysis of early cellular response was (for all of the stimuli we used) the basophil threshold sensitivity approach with the construction of complete dose response curves and not only with limited concentrations, as in our previous study when monitoring HBV immunotherapy in children at the same time points [20]. In the previous study, the basophils were stimulated with a maximum concentration of anti-FcεRI mAbs (550 ng/ml) and with 1 and 0.1 µg/ml of venom and thus only insignificant basophil changes before the first MD were evident. Comparable insignificant findings would be found in the current report if only those limited concentrations were analyzed.

The foundation of specific immunotherapy is based on the allergen specificity of this treatment. Our challenging results suggest that, in contrast to long-term VIT, which induces venom-specific changes, the effect of short-term VIT may be venom-nonspecific. Similar nonspecific effect of short-term desensitization was observed as decreased peripheral leukocyte histamine release, specifically to desensitizing allergen as well as nonspecifically to other co-sensitizing allergens [12]–[14], [16], [17]. Currently, we do not know the clinical relevance of these early basophil changes and whether they are also clinically relevant for co-sensitizing allergens. Nevertheless, the anti-IgE treatment model demonstrated that a reduction of symptoms occurs when the basophils become comparably desensitized, as shown in our model [35], [36]. In addition, this study was not designed to assess whether FcεRI-mediated basophil desensitization appears in the later course of VIT because we primarily focused on the point before obtaining the first MD of 100 µg of venom, which is a hallmark for the long maintenance period.

In summary, this study showed that early VIT-induced suppression of cellular FcεRI pathway results in comparable basophil desensitization to both VIT-specific and VIT-nonspecific venom. Furthermore this nonspecific desensitization was evident in case of other co-sensitizations (i.e., grass pollen) and with HDM passive IgE sensitization. Thus, further preferably larger multicentre studies are warranted a) to examine the clinical relevance of this early cellular desensitization, not only concerning VIT allergen, but also other co-sensitizing allergens; b) to address the later course of these changes; and c) to examine in detail the exact mechanism of this cellular shift because this would allow the development of novel interventions for promoting or monitor the silencing of basophil FcεRI pathway.
